# Associations between Perinatal Sleepiness and Breastfeeding Intentions and Attitudes and Infant Feeding Behaviors and Beliefs

**DOI:** 10.3390/nu15153435

**Published:** 2023-08-03

**Authors:** Tayla von Ash, Anna Alikhani, Katherine M. Sharkey, Paola Solano, Melanie Morales Aquino, Patricia Markham Risica

**Affiliations:** 1 Department of Behavioral and Social Science, Brown School of Public Health, Providence, RI 02903, USA; anna_alikhani@brown.edu; 2 Center for Health Promotion and Health Equity, Brown School of Public Health, Providence, RI 02903, USA; 3 Department of Psychiatry & Human Behavior, Alpert Medical School of Brown University, Providence, RI 02906, USA; katherine_sharkey@brown.edu; 4 Department of Medicine, Alpert Medical School of Brown University, Providence, RI 02903, USA; 5 Rhode Island Hospital, 593 Eddy Street, Providence, RI 02903, USA; 6 Brown University, Providence, RI 02912, USA; paola_solano@alumni.brown.edu (P.S.); melanie_morales_aquino@brown.edu (M.M.A.)

**Keywords:** sleep, sleepiness, breastfeeding, breastfeeding intentions, breastfeeding attitudes, breastfeeding behaviors, infant feeding

## Abstract

Breastfeeding rates fall short of public health goals, but barriers are poorly understood. We examined whether excessive sleepiness during pregnancy and the postpartum period was associated with breastfeeding intentions, attitudes, initiation, and continuation in a tobacco-exposed sample participating in a randomized controlled trial to reduce smoke exposure (n = 399). We used the Epworth Sleepiness Scale (ESS) to examine associations between excessive sleepiness in early (12–16 weeks gestation) and late (32 weeks gestation) pregnancy and at 6 months postpartum, with breastfeeding attitudes using the Mitra index, intentions, initiation, and continuation, as well as other infant feeding practices using the Infant Feeding Questionnaire. Logistic regression models adjusted for age, racial/ethnic identity, parity, marital status, and maternal education showed that excessive sleepiness in late pregnancy was associated with less favorable attitudes toward breastfeeding. In addition, in unadjusted models, excessive sleepiness at 6 months postpartum was associated with less of a tendency to use feeding to calm a fussy infant. Excessive sleepiness was not associated with intent, initiation, or continuation of breastfeeding. Assessing excessive sleepiness in late pregnancy may assist in identifying individuals with negative attitudes to breastfeeding and lead to novel approaches to promoting breastfeeding in populations with lower breastfeeding rates.

## 1. Introduction

Breastfeeding is associated with a decreased risk of infant and child respiratory infections [1,2,3,4], asthma [5,6], childhood obesity [7,8,9], metabolic syndrome [10], cardiovascular disease (CVD) risk [11,12,13], childhood cancers [14,15], and infant mortality [16,17]. Breastfeeding also has benefits for lactating persons, as it reduces the risk of CVD, diabetes, and certain cancers [4,18,19]. Additionally, breastfeeding can yield psychological benefits, as it fosters bonding and increases self-confidence in new parents [20]. Given the plethora of benefits of breastfeeding, the World Health Organization (WHO) and the American Academy of Pediatrics (AAP) recommend that infants be exclusively breastfed for their first six months of life [4,21]. However, it is estimated that less than three-fifths of U.S. infants are breastfeeding at 6 months, with approximately one-fourth exclusively breastfeeding [22]. Moreover, persistent disparities in breastfeeding exist, with lower breastfeeding rates among low-income and racial and ethnic minority groups compared to non-Hispanic White samples [23,24,25]. Recognized barriers to successful breastfeeding include personal, medical, economic, societal, and cultural obstacles [26,27], but a better understanding of the factors that influence breastfeeding, especially in high-risk samples, may help to increase breastfeeding rates.

One factor that may be associated with breastfeeding but has not been thoroughly researched is maternal sleep. A few studies examining how breastfeeding impacts maternal sleep duration found that exclusive breastfeeding is associated with decreased sleep duration [28], while others have found it to be associated with increased sleep duration [29,30,31]. Results from studies examining the relationship between daytime symptoms of fatigue and breastfeeding are also mixed. Postpartum fatigue is independent of the feeding method in some studies [32,33,34], yet others have shown that fatigue is negatively associated with breastfeeding and is often a reason for early breastfeeding cessation [19,35,36,37]. Sleepiness is a phenomenon that is related to, but distinct from, fatigue, and often refers to one’s actual tendency to fall asleep during daytime activities [38]. While we could not find any published studies that have examined the association between sleepiness and breastfeeding, we hypothesize that maternal sleepiness is negatively associated with breastfeeding given that breastfeeding often takes longer than bottle feeding and that falling asleep while breastfeeding could pose a danger to infants (e.g., if an infant is dropped or is at increased risk for accidental suffocation) [39].

The purpose of this secondary data analysis is to explore associations between maternal sleepiness and breastfeeding. Beyond being the first to examine this relationship, this analysis addresses additional gaps in the breastfeeding sleep literature. First, while one study found that poor sleep quality during pregnancy is associated with lower rates of breastfeeding initiation [27], most studies have focused on the postpartum period. In addition to examining associations between sleepiness and breastfeeding behavior in the postpartum period, this analysis examines the relationship between sleepiness and breastfeeding intentions and attitudes, key predictors of breastfeeding behaviors [20,40,41,42,43,44], during early and late pregnancy. We also examine how sleepiness impacts feeding practices and beliefs beyond just those related to breastfeeding. Finally, the analysis is conducted in a high-risk sample of low-income tobacco smoke-exposed women who are often underrepresented in the breastfeeding literature despite being less likely to initiate breastfeeding [45,46,47] and breastfeeding for shorter durations than nonsmokers [47,48,49]. Moreover, individuals who are smoke-exposed, particularly to tobacco smoke, have poorer sleep quality than those not exposed [50,51], and smoke exposure can impact infant sleep, further impacting parent sleep [52,53].

## 2. Methods

### 2.1. Study Design

This secondary analysis used data from Baby’s Breath, a tailored video intervention tested in a randomized control trial (NCT00142623), aimed to reduce smoke exposure among pregnant women and their infants. For this study, we used sleepiness ratings collected at three time points: Early pregnancy (12–16 weeks gestation), late pregnancy (32 weeks gestation), and 6 months postpartum, as well as breastfeeding intentions and attitudes collected at both pregnancy time points, breastfeeding initiation upon hospital discharge after delivery, breastfeeding behavior at 3 and 6 months postpartum, and infant feeding practices at 6 months postpartum. 

Participants, who were recruited from prenatal clinics that largely serve low-income families in the Providence, RI area, were eligible if they spoke English, were at least 18 years of age, smoke-exposed (i.e., currently smoking, recently quit, or exposed to environmental tobacco smoke), experiencing a normal singleton pregnancy, and not more than 16 weeks pregnant at the time of recruitment. Participants also needed to have access to a working telephone and a VCR/DVD player. Details about the intervention can be found in Risica et al. (2017) [54], but briefly, participants were randomized to receive informational materials during pregnancy and the first six months of the postpartum period aimed at either smoking cessation and avoidance or other healthy pregnancy topics; information regarding infant feeding was not part of the core content and was identical for both experimental groups. The analytic sample for the present study includes 399 participants who had complete data for the variables of interest in our analyses (i.e., sleepiness, breastfeeding intentions, attitudes, and behavior, and infant feeding practices and beliefs).

### 2.2. Data Collection and Measures

Computer-assisted telephone interview (CATI) surveys were utilized to collect data at different time points during gestation and postpartum. Sociodemographic characteristics, which were collected at study enrollment between 12 and 16 weeks gestation, included age, self-reported race/ethnicity, level of education, employment status, annual household income, marital status, and parity.

Excessive sleepiness: Sleepiness is defined as the tendency to fall asleep or the need to resist falling asleep during usual daytime activities. Participants reported their subjective sleepiness at 12–16 weeks gestation (early pregnancy), 32 weeks gestation (late pregnancy), and 6 months postpartum using the Epworth Sleepiness Scale (ESS) [55]. The ESS is a widely used measure that assesses the likelihood of falling asleep during eight situations: Sitting and reading, watching television, sitting inactive in a public space, as a passenger in a car for an hour without a break, lying down to rest in the afternoon, sitting and talking to someone, sitting quietly after lunch without alcohol, and in a car while stopped for a few minutes in traffic. Responses, which include no chance of dozing off (0), slight chance of dozing off (1), moderate chances of dozing off (2), and a high chance of dozing off (3), are summed to generate a total score, with higher scores signifying increased sleepiness. Scores for each item are added together to calculate a total ESS score (which can range from 0–24). As a score of 10 or higher indicates clinically significant daytime sleepiness, we used this cut-off to create a dichotomous variable for excessive sleepiness. The ESS has been demonstrated to be a valid and reliable measure of daytime sleepiness in many populations including pregnant women [56,57].

Breastfeeding intentions: Breastfeeding intentions were assessed in early and late pregnancy (i.e., 12–16 and 32 weeks gestation). Participants indicated how they planned to feed their baby by selecting from the following categories: “intend to breastfeed”, “intend to breastfeed and formula feed”, “intend to formula feed”, or “undecided”. This variable was dichotomized as “not considering breastfeeding” if they did not intend to breastfeed, or “considering breastfeeding” if they considered or intended to breastfeed (either exclusively or partially) or were undecided. We included those who were undecided in the considering breastfeeding group as 57% of the undecided participants in this sample went on to initiate breastfeeding [58]. 

Breastfeeding attitudes: Breastfeeding attitudes were assessed in early and late pregnancy using the Mitra index [59]. The Mitra index includes 18 items assessing breastfeeding-related thoughts and attitudes and includes 5 subscales: (1) Knowledge, (2) self-efficacy, (3) embarrassment barriers, (4) social and time barriers, and (5) social support barriers. In prior research on this dataset, the measure was modified from its original true/false response options to create a five-point Likert scale ranging from “agree a lot” to “disagree a lot” [58]. Responses were allocated numerical values ranging from −2 to +2, with +2 representing the strongest response in the direction favoring breastfeeding support. A global index score is created by taking the average of the responses from the individual items, with a higher overall score indicating more favorable breastfeeding-related thoughts and attitudes. Cronbach’s alpha for the Mitra index in this sample was 0.76, demonstrating strong internal consistency [58].

Breastfeeding behavior: Participants reported on a check-in call soon after delivery if they had initiated breastfeeding. At 3 and 6 months postpartum, participants reported if they were exclusively breastfeeding, mixed feeding (breastfeeding and formula feeding), or formula feeding. Breastfeeding at 3 and 6 months was dichotomized into “any breastfeeding” or “exclusive formula feeding”.

Other infant feeding practices and beliefs: We used the Infant Feeding Questionnaire (IFQ) to assess maternal feeding practices and beliefs beyond breastfeeding [60]. Administered at 6 months, the IFQ consists of 20 items (12 on feeding practices and 8 on beliefs) and includes 7 subscales: (1) Concern about infant undereating or becoming underweight, (2) concern about infant’s hunger, (3) awareness of infant’s hunger and satiety cues, (4) concern about infant overeating or becoming overweight, (5) feeding infant on a schedule, (6) using food to calm infant’s fussiness, and (7) social interaction with the infant during feeding [60]. Responses are allocated numerical values ranging from 0 to 5, with higher scores indicating a higher frequency of maternal feeding practices and higher agreement with the maternal belief statements. Subscale scores are created by averaging the responses from the individual items in each subscale. Cronbach’s alphas for each factor in this sample were similar to those reported by Baughcum et al. (2001) [60,61].

### 2.3. Statistical Analysis

Descriptive statistics were calculated for sociodemographic characteristics and feeding-related variables. We examined bivariate associations between each sociodemographic characteristic and sleepiness scores using analysis of variance (ANOVA). Likewise, we examined bivariate associations between each sociodemographic characteristic and the breastfeeding variables. Variables that were associated with either sleepiness or breastfeeding at a modest significance level (*p* < 0.2) were included as covariates in subsequent multivariate analyses. This included age, race/ethnicity, parity, marital status, and maternal education.

To examine cross-sectional and longitudinal associations between excessive sleepiness (as a predictor) during early and late pregnancy and at 6 months postpartum, with breastfeeding intentions, initiation, and behavior at 3 and 6 months (all dichotomous outcomes), we constructed a series of logistic regression models. To examine cross-sectional associations between excessive sleepiness during early and late pregnancy (predictor) with breastfeeding attitudes during pregnancy (continuous outcomes), we constructed a series of linear regression models. Likewise, we used linear regression models to examine cross-sectional associations between excessive sleepiness at 6 months postpartum (predictor) with infant feeding practices and beliefs beyond breastfeeding (continuous outcomes). Data were analyzed using the statistical software package STATA and statistical significance was set at the 0.05 level.

## 3. Results

### 3.1. Sociodemographic Characteristics

Participants’ average age was 24 years old (SD 5.02), and they reported diverse racial/ethnic backgrounds as shown in Table 1: 41% non-Hispanic White, 27% Hispanic, 13% non-Hispanic Black, and 19% other or multiracial. Included in the other or multiracial group were 33 participants identifying as American Indian/Alaska Native, 15 as Asian, 2 as Native Hawaiian/Pacific Islander, and 41 as multiracial. Approximately one-third (36%) of the sample had not received a high school diploma and nearly half (47%) were unemployed. Likewise, nearly half (48%) had an annual household income of less than $10,000 USD. As for family structure, approximately half of the participants (47%) were either married or cohabitating with a partner and half (50%) were multiparous. 

### 3.2. Excessive Sleepiness

Average ESS was 6.6 (SD 4.1) in early pregnancy, 6.0 (3.8) in late pregnancy, and 4.1 (SD 3.7) at 6 months postpartum. The percentage of women who reported excessive daytime sleepiness, defined as an ESS score ≥ 10, was 20% in early pregnancy, 18% in late pregnancy, and 9% at 6 months postpartum. Excessive sleepiness was not related to tobacco use at baseline nor group assignment in the parent clinical trial. 

### 3.3. Breastfeeding Intent, Initiation and Continuation

**Figure 1:**
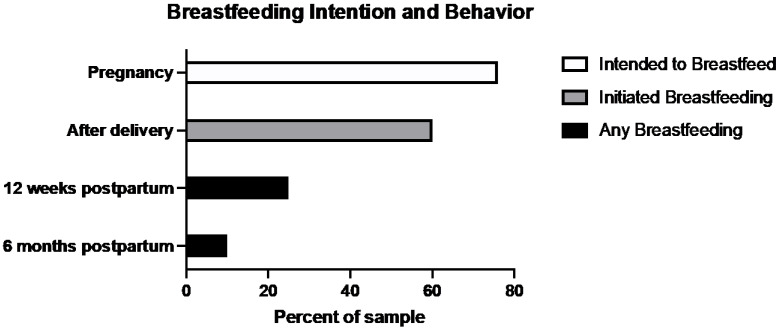
Sample breastfeeding intentions, initiation, and continuation rates.

As shown in Figure 1, most participants intended to breastfeed, with 76% reporting that they were considering breastfeeding at both the early and late pregnancy timepoints as shown in Table 2. Similarly, the majority, 60%, initiated breastfeeding after birth. At 3 months postpartum, 25% of the sample reported any breastfeeding, and by 6 months, only 10% of mothers in the sample reported any breastfeeding.

### 3.4. Associations between Excessive Sleepiness and Breastfeeding Intent, Initiation, and Continuation

There were no significant cross-sectional associations between the presence of excessive sleepiness in early (12–16 weeks gestation) or late (32 weeks gestation) pregnancy and breastfeeding intention, nor did excessive sleepiness at either pregnancy time point predict the initiation of breastfeeding immediately after delivery or continuation of breastfeeding at 3 or 6 months postpartum. Additionally, we did not observe a cross-sectional association between excessive sleepiness and continued breastfeeding at 6 months postpartum (see Table 3). 

### 3.5. Associations between Excessive Sleepiness in Pregnancy and Breastfeeding Attitudes

We did not observe any cross-sectional associations between excessive sleepiness in early pregnancy and a favorable disposition toward breastfeeding. On the other hand, during late pregnancy, participants who reported clinically significant sleepiness had overall less favorable attitudes toward breastfeeding (*β*: −0.19, [95% CI: −0.36, −0.02], *p* = 0.02) compared to those who did not report sleepiness, even after controlling for age, race/ethnicity, parity, marital status, and maternal education. They also had lower scores for embarrassment barriers indicating greater perceived barriers related to breastfeeding embarrassment (see Table 4). 

### 3.6. Associations between Excessive Postpartum Sleepiness and Other Infant Feeding Practices

When examining associations between sleepiness at 6 months postpartum and concurrent infant feeding practices and beliefs (Table 5), we found that excessive sleepiness was associated with lower reporting of using food to calm infant fussiness. However, the association was significant in the unadjusted model only, with mothers with higher sleepiness scores at 6 months less likely to report using food to calm infant fussiness (*β*: −0.39, [95% CI: −0.77, −0.02], *p* = 0.02).

## 4. Discussion

The goal of this exploratory analysis was to examine relationships between subjectively reported excessive sleepiness during early and late pregnancy and at 6 months postpartum with breastfeeding intentions and behavior, as well as attitudes and beliefs about other aspects of infant feeding. We found that excessive sleepiness in late pregnancy was associated with less favorable attitudes towards breastfeeding. Specifically, participants with Epworth Sleepiness Scale (ESS) scores ≥ 10 had lower scores on the Mitra Index after adjusting for age, racial/ethnic identity, parity, marital status, and maternal education. Unadjusted models showed that those with excessive sleepiness were more likely to report obstacles related to time and social constraints (e.g., It would take too much time for me to breastfeed my baby) and embarrassment factors (e.g., I would feel shy breastfeeding outside my home). We did not find an association between sleepiness and breastfeeding intentions or behavior.

Between 18 and 20% of our sample reported excessive daytime sleepiness in pregnancy, which is similar to rates in other studies [62,63,64]. For example, in a community-based sample assessed immediately after delivery (n = 1000), Bourjeily and colleagues found elevated ESS scores in 18% of participants and showed that ESS scores were higher among patients with planned Cesarean deliveries compared to those who had uncomplicated vaginal deliveries [64]. Moreover, those who reported snoring were more likely to have clinically significant sleepiness as measured by ESS. Similarly, excessive sleepiness was reported by 22% of a sample surveyed in the late third trimester and was more common among participants who worked outside the home [62]. Our study did not examine potential sources of excessive daytime sleepiness among our participants; however, based on prior studies and factors related to time and logistical barriers in our sample, we posit that those with excessive daytime sleepiness in late pregnancy may have viewed breastfeeding as a less favorable option because of competing responsibilities or the presence of health concerns.

At 6 months postpartum, our unadjusted model showed that participants with excessive sleepiness had a lower tendency to use feeding to soothe their fussy infants. More granular data should be collected to confirm and explain this intriguing finding. In the meantime, given evidence of emotional congruity in the mother–infant dyad [65], we postulate that sleepy parents may attribute infant fussiness to sleepiness rather than hunger, thereby decreasing the likelihood of responding to the infant’s distress by feeding [66]. This hypothesis is also consistent with the trend toward lower sensitivity to hunger cues among sleepy mothers compared to those that were less sleepy. Another explanation for this finding is that participants who reported excessive sleepiness may have had less desire to engage in more demanding child-rearing activities. By 6 months, most infants in our sample were not being breastfed. Thus, feeding a fussy infant would require the preparation of a bottle or other food. It is possible that those with clinically significant sleepiness would be less inclined to prepare food to soothe their infant before trying other techniques.

We did not find an association between excessive sleepiness during early or late pregnancy and intention to breastfeed or initiation of breastfeeding in our sample. Moreover, excessive sleepiness in pregnancy did not predict continued breastfeeding at 3 or 6 months postpartum. Rates of continued breastfeeding were lower in our sample than have been reported in other studies in community samples [67,68], consistent with the observation that tobacco-exposed women have lower breastfeeding rates [45,46,47,48]. Thus, we may not have had adequate statistical power to show a relationship between sleepiness and breastfeeding behaviors in this sample. In a sample at risk for developing postpartum depression, we recently showed that greater objectively measured sleep disturbance in pregnancy was associated with a higher likelihood of not initiating or continuing breastfeeding [27]. Future work should aim to disentangle how subjective and objective sleep in the perinatal period contributes to feeding behaviors. Future studies should also aim to measure and adjust for potential confounders beyond those we considered (e.g., stress, depression, and anxiety), which could impact both sleepiness and breastfeeding.

This analysis has both strengths and limitations. Our findings add to a very small body of literature examining associations between sleep during pregnancy and breastfeeding behavior, and to our knowledge, this is the first study to examine associations with breastfeeding attitudes and intent, and infant feeding practices and beliefs beyond those related to breastfeeding. Both sleep and breastfeeding variables (i.e., attitudes, intentions, and behaviors) were assessed at multiple timepoints, allowing us to examine longitudinal associations in addition to cross-sectional associations. However, the only postpartum measure of sleepiness we had was at 6 months postpartum. Future studies should aim to assess sleepiness at hospital discharge and earlier in the postpartum period (e.g., 3 months) because sleepiness during these important timepoints (i.e., before the feeding method is solidified and while infant night wakings are frequent) might have a stronger impact on breastfeeding behaviors. Another limitation of this secondary analysis is that subjective sleepiness was the only sleep-related variable that was measured. The multiple sleep latency test is an objective measure of the rapidity of latency to sleep onset using polysomnography and is considered the gold standard measure of daytime sleepiness [69]. This measure is not practical, however, in all settings, and subjectively reported daytime sleepiness measures correlate with objective assessments. That said, other characteristics of sleep (e.g., duration and quality) may be more strongly related to infant feeding decisions. Finally, the results of this study may not be generalizable to pregnant women more broadly, as the sample was comprised of low-income, smoke-exposed women from southern New England, and the relationship between sleepiness and infant feeding decisions may differ for this group. For example, environmental tobacco exposure has been shown to be associated with daytime sleepiness [70], including among pregnant women [51], and people who are cigarette smokers can experience trouble falling and staying asleep as well as greater daytime sleepiness [71]. However, while a study of more than 4000 adults found that ever-smokers exhibited differences in several sleep disturbance measures compared to never-smokers, they did not differ in sleepiness [72]. We also did not find an association between smoking status and sleepiness in this sample so did not control for it in the multivariate analyses. Nonetheless, the relationship between smoking status, sleepiness, and breastfeeding warrants further examination as many smokers attempt to quit smoking during pregnancy, and sleep disturbances have been documented during smoking cessation [73].

## 5. Conclusions

Understanding barriers to breastfeeding is important because breastfeeding is associated with numerous health benefits, both for infants and lactating persons. This study found that mothers with excessive sleepiness in late pregnancy had less favorable attitudes towards breastfeeding. Assessing excessive sleepiness in late pregnancy may thus assist in identifying individuals with negative attitudes to breastfeeding and lead to novel approaches to promoting breastfeeding, especially in populations with lower breastfeeding rates. We also found that mothers with excessive sleepiness at 6 months postpartum were less likely to use feeding to calm a fussy infant, suggesting that clinically significant sleepiness may impact how parents respond to their infants, though more research is needed to confirm this finding. Nonetheless, this study expands the relatively small and narrow literature on maternal sleep and infant feeding plans and behavior and demonstrates that associations are present prior to giving birth.

## Figures and Tables

**Table 1:** Sample sociodemographic characteristics with baseline sleepiness scores.

	n (%)
Overall	399 (100)
Age	
<21 years	112 (28)
21–25 years	151 (38)
>25 years	136 (34)
Race/ethnicity	
White, non-Hispanic	160 (41)
Black, non-Hispanic	53 (13)
Hispanic	109 (27)
Other/more than one ^1^	77 (19)
Education	
Less than high school	144 (36)
High-school graduate/GED	142 (36)
Some college/technical school	109 (28)
Employment status	
Full time	87 (22)
Part time	90 (23)
Not employed	186 (47)
Other	35 (9)
Annual household income	
<$10,000 USD	165 (48)
$10,000–30,000 USD	129 (37)
>$30,000 USD	51 (15)
Marital status	
Married or cohabitating	184 (47)
Not married or cohabitating	212 (54)
Parity	
Previous live births	185 (50)
No previous live births	184 (50)

^1^: Includes 33 participants identifying as American Indian/Alaska Native, 15 as Asian, 2 as Native Hawaiian/Pacific Islander, and 41 as multiracial.

**Table 2:** Sample feeding-related characteristics.

	n (%)
Breastfeeding intentions during pregnancy	
Feeding intentions at baseline (12–16 weeks gest.)	
Considering breastfeeding	277 (76)
Not considering breastfeeding	88 (24)
Feeding intentions at 32 weeks gest.	
Considering breastfeeding	303 (76)
Not considering breastfeeding	96 (24)
Breastfeeding attitudes during pregnancy	
Breastfeeding attitudes at baseline (12–16 weeks gest.)	
Overall Mitra index ^1^	0.65 (0.64)
Knowledge subscale ^1^	0.75 (0.84)
Self-efficacy subscale ^1^	0.87 (1.02)
Embarrassment barriers subscale ^1^	−0.12 (1.12)
Social and time barriers subscale ^1^	0.95 (0.84)
Social support barriers subscale ^1^	0.90 (0.86)
Breastfeeding attitudes at 32 weeks gest.	
Overall Mitra index ^1^	0.77 (0.64)
Knowledge subscale ^1^	0.92 (0.81)
Self-efficacy subscale ^1^	0.89 (1.04)
Embarrassment barriers subscale ^1^	0.04 (1.17)
Social and time barriers subscale ^1^	1.08 (1.08)
Social support barriers subscale ^1^	1.01 (0.87)
Breastfeeding behavior	
Breastfeeding initiation	
Yes (exclusive or partial breastfeeding)	209 (61)
No (formula-feeding only)	134 (39)
Breastfeeding at 12 weeks	
Yes (exclusive or partial breastfeeding)	82 (25)
No (formula-feeding only)	244 (75)
Breastfeeding at 6 months	
Yes (exclusive or partial breastfeeding)	33 (10)
No (formula-feeding only)	310 (90)
Infant feeding practices and beliefs at 6 months	
Concern About Infant Undereating or Becoming Underweight ^1^	3.24 (0.76)
Concern About Infant’s Hunger ^1^	2.55 (1.14)
Awareness of Infant’s Hunger and Satiety Cues ^1^	0.19 (0.41)
Concern About Infant Overeating or Becoming Overweight ^1^	3.19 (0.86)
Feeding Infant on a Schedule ^1^	2.55 (1.20)
Using Food to Calm Infant’s Fussiness ^1^	2.10 (1.05)
Social Interaction with the Infant During Feeding ^1^	0.61 (0.72)

^1^: mean (standard deviation presented as opposed to n (%).

**Table 3:** Associations between excessive sleepiness during pregnancy and at 6 months postpartum with breastfeeding intentions and behavior—using cut-off point of 10.

	Unadjusted OR [95% CI]	Adjusted ^1^ OR [95% CI]
Excessive sleepiness early pregnancy with…		
Breastfeeding intention (12–16 weeks)	1.32 [0.70, 2.46]	1.47 [0.71, 3.06]
Breastfeeding initiation	1.01 [0.59, 1.75]	1.04 [0.55, 1.97]
Breastfeeding at 12 weeks	0.95 [0.46, 1.95]	0.74 [0.33, 1.68]
Breastfeeding at 6 months	0.90 [0.34, 2.34]	0.95 [0.32, 2.83]
Excessive sleepiness late pregnancy with...		
Breastfeeding intention (32 weeks)	0.86 [0.48, 1.54]	0.85 [0.44, 1.66]
Breastfeeding initiation	0.86 [0.48, 1.53]	0.93 [0.47, 1.83]
Breastfeeding at 12 weeks	1.56 [0.73, 3.33]	1.55 [0.68, 3.55]
Breastfeeding at 6 months	1.23 [0.46, 3.25]	0.92 [0.28, 3.01]
Ex. sleepiness at 6 months postpartum with...		
Breastfeeding at 6 months	1.07 [0.29, 3.94]	0.44 [0.06, 3.65]

^1^: adjusted for age, race/ethnicity, parity, marital status, and maternal education.

**Table 4:** Associations between excessive sleepiness and breastfeeding attitudes during pregnancy.

	Unadjusted β [95% CI]	Adjusted ^1^ β [95% CI]
Early pregnancy (12–16 weeks gestation)		
Overall Mitra index	0.03 [−0.13, 0.18]	0.03 [−0.13, 0.20]
Mitra index subscales		
Knowledge	0.06 [−0.14, 0.27]	0.11 [−0.12, 0.33]
Self-efficacy	−0.02 [−0.27, 0.23]	0.06 [−0.21, 0.32]
Embarrassment barriers	0.15 [−0.12, 0.43]	0.12 [−0.18, 0.41]
Social and time barriers	−0.10 [−0.30, 0.11]	−0.12 [−0.34, 0.10]
Social support barriers	0.03 [−0.18, 0.24]	0.03 [−0.19, 0.25]
Late Pregnancy (32 weeks gestation)		
Overall Mitra index	−0.18 [−0.34, −0.01] *	−0.19 [−0.36, −0.02] *
Mitra index subscales		
Knowledge	−0.02 [−0.23, 0.19]	0.02 [−0.20, 0.24]
Self-efficacy	−0.25 [−0.52, 0.01]	−0.19 [−0.47, 0.09]
Embarrassment barriers	−0.40 [−0.70, −0.10] *	−0.55 [−0.87, −0.24] *
Social and time barriers	−0.21 [−0.41, −0.01] *	−0.19 [−0.40, 0.03]
Social support barriers	0.02 [−0.21, 0.24]	0.03 [−0.20, 0.26]

*: *p* < 0.05; ^1^: adjusted for age, race/ethnicity, parity, marital status, and maternal education.

**Table 5:** Associations between excessive sleepiness and infant feeding practices and beliefs at 6 months postpartum.

	Unadjusted *β* [95% CI]	Adjusted ^1^ *β* [95% CI]
Concern About Infant Undereating or Becoming Underweight	−0.11 [−0.38, 0.17]	−0.12 [−0.41, 0.17]
Concern About Infant’s Hunger	0.01 [−0.41, 0.42]	−0.08 [−0.34, 0.51]
Awareness of Infant’s Hunger and Satiety Cues	−0.09 [−.24, 0.05]	−0.13 [−0.28, 0.03]
Concern About Infant Overeating or Becoming Overweight	−0.17 [−0.49, 0.14]	−0.19 [−0.52, 0.14]
Feeding Infant on a Schedule	0.15 [−.28, 0.59]	0.15 [−0.29, 0.59]
Using Food to Calm Infant’s Fussiness	−0.39 [−0.77, −0.02] *	−0.33 [−0.73, 0.06]
Social Interaction with the Infant During Feeding	−0.09 [−0.35, 0.17]	−0.16 [−0.42, 0.11]

*: *p* < 0.05; ^1^: adjusted for age, race/ethnicity, parity, marital status, and maternal education.

## Data Availability

The data presented in this study are available on request from the corresponding author.
